# Altered Functional Brain Network Structure between Patients with High and Low Generalized Anxiety Disorder

**DOI:** 10.3390/diagnostics13071292

**Published:** 2023-03-29

**Authors:** Xuchen Qi, Jiaqi Fang, Yu Sun, Wanxiu Xu, Gang Li

**Affiliations:** 1 Department of Neurosurgery, Sir Run Run Shaw Hospital, Zhejiang University School of Medicine, Hangzhou 310000, China; 2 Department of Neurosurgery, Shaoxing People’s Hospital, Shaoxing 312000, China; 3 College of Engineering, Zhejiang Normal University, Jinhua 321004, China; 4 Key Laboratory for Biomedical Engineering of Ministry of Education of China, Department of Biomedical Engineering, Zhejiang University, Hangzhou 310000, China; 5 College of Mathematical Medicine, Zhejiang Normal University, Jinhua 321004, China

**Keywords:** generalized anxiety disorder (GAD), electroencephalogram (EEG), functional brain network, functional connectivity (FC), small world

## Abstract

To investigate the differences in functional brain network structures between patients with a high level of generalized anxiety disorder (HGAD) and those with a low level of generalized anxiety disorder (LGAD), a resting-state electroencephalogram (EEG) was recorded in 30 LGAD patients and 21 HGAD patients. Functional connectivity between all pairs of brain regions was determined by the Phase Lag Index (PLI) to construct a functional brain network. Then, the characteristic path length, clustering coefficient, and small world were calculated to estimate functional brain network structures. The results showed that the PLI values of HGAD were significantly increased in alpha2, and significantly decreased in the theta and alpha1 rhythms, and the small-world attributes for both HGAD patients and LGAD patients were less than one for all the rhythms. Moreover, the small-world values of HGAD were significantly lower than those of LGAD in the theta and alpha2 rhythms, which indicated that the brain functional network structure would deteriorate with the increase in generalized anxiety disorder (GAD) severity. Our findings may play a role in the development and understanding of LGAD and HGAD to determine whether interventions that target these brain changes may be effective in treating GAD.

## 1. Introduction

Generalized anxiety disorder (GAD) is characterized by excessive anxiety and uncontrollable worry about a variety of topics and persists for a long time [1,2,3,4]. Other typical characteristics include autonomic nervous dysfunction, muscle tension, and concentration difficulties. These lead to distress or impairments in daily life and functioning [5]. In recent years, many neuropsychological studies have pointed out that individuals with anxiety have an increased risk of cognitive impairment, and the decline of these cognitive functions will lead to increased anxiety symptoms, forming a vicious cycle [4,6,7]. With the development of society, its incidence rate is increasing year by year. The prevalence of GAD for each year is 3.1% in the United States of America. For China and the United Kingdom, the rates are 5.3% and 6%, respectively [3,4].

A high level of GAD (HGAD) and low level of GAD (LGAD) refer to the severity of symptoms in people with generalized anxiety disorder. Specifically, HGAD refers to severe anxiety symptoms that may significantly negatively affect a person’s daily life. On the other hand, LGAD refers to mild anxiety symptoms that may not greatly impact a person’s daily life. Generally, treatment for HGAD requires greater intervention and may involve medication and psychotherapy. On the other hand, treatment for LGAD may only require psychotherapy, such as cognitive behavioral therapy or general psychotherapy. LGAD patients may also be able to alleviate their symptoms through self-management techniques such as meditation, breathing techniques, or exercise to reduce anxiety. Treatments for different levels of GAD are different. If the patients are not treated promptly, its prognosis will be poor. To select the proper treatment and evaluate the effect of treatment, it is very important to understand the difference in severity. The severity of GAD symptoms has been categorized according to the Hamilton Anxiety Rating Scale (HAMA) [8] and the doctor’s diagnosis. However, there is no corresponding evidence of the neurodynamics between different levels of GAD. What remains unclear, as well, is whether there is a relationship between the severity of GAD and cerebral region functional activity. 

Various electrophysiological techniques, such as positron emission tomography, magnetic resonance imaging, and electroencephalography (EEG), have been widely used to measure neuronal activity [9,10,11,12]. Among them, EEG technology has the advantages of low cost, good portability, and strong portability [13,14,15,16]. The electrical signals sent by the human brain through the EEG instrument are captured by placing electrodes on the scalp through EEG technology to record brain activity [17,18,19,20]. The potential of the brain cell group is used as the vertical axis, and the time is used as the horizontal axis. It is displayed in the form of a curve, also known as an EEG. Brain waves can be decomposed into waves ranging from small to large in frequency, which are collectively referred to as biological rhythm waves [21]. They could be classified with the frequency range as delta, theta, alpha, beta, and gamma rhythms [18,20,22,23,24,25]. For GAD, it has been proven that the beta rhythm has significant changes in power spectrum density (PSD), complexity, and FC based on EEG signals [4]. 

GAD with different levels may have differences involving different brain regions. However, the structure and function of the brain’s neural networks have not been studied due to great limitations. Different brain regions have their own specific functions. These regions need to cooperate; otherwise, they cannot complete a task alone [26]. Therefore, cooperation between different areas is required to form functional brain networks. A functional brain network is composed of coordination among various elements in the system [27,28,29,30,31,32]. To some extent, FC can reflect the functional interaction between different brain regions. It has been widely employed [27,29,33].

Measures such as the clustering coefficient, path length, and efficiency are usually used to characterize the system of functional brain networks at local and global levels [34,35]. When the average path length is shorter and the average clustering coefficient is larger, the information processing and transmission is faster [29]. It can be effectively used to evaluate the level of GAD and analyze the neurodynamic mechanism of GAD. The small-world network model is well used to quantify the topological structure and dynamic characteristics of a brain functional network [36,37]. In addition, the optimal connection mode of the brain for various task activities could be ensured by the brain functional network with small-world characteristics by balancing and optimizing the process of functional separation and integration. 

Thus, it is important to establish a functional brain network model based on EEG signals to analyze different levels of GAD. Network characteristics, namely the feature path length, clustering coefficient, and small-world network model, are selected to deeply analyze the functional structures of GAD patients at different levels.

## 2. Materials and Methods

### 2.1. Participants

The data were obtained from 51 GAD patients enrolled at the Huzhou Third People’s Hospital and Shaoxing People’s Hospital. Each patient met the diagnostic criteria in the fourth revision of the Diagnostic and Statistical Manual of Mental Disorders (DSM-V); the age ranged from 27 to 58 years old and their scores of HAMA were greater than 17. Meanwhile, all the patients were required to be right-handed, with no alcohol and substance abuse, no history of brain damage, no other organic mental disorders (such as Alzheimer’s), no schizophrenia or other psychiatric disorders, no mood disorders (such as depression, bipolar disorder), and no mental disorders caused by psychoactive substances or non-addictive substances that may impair brain function. In addition, they were required to be non-illiterate so as to complete the HAMA scale assessment independently. One day before the experiment, each patient was required to take normal rest, with no alcohol or drugs and no strong tea or coffee. 

According to the HAMA scores, GAD patients were divided into two groups, HGAD and LGAD. The HAMA score for HA was no less than 29, and the HAMA score for LA was less than 29. The demographic and clinical characteristics of LGAD and HA are shown in Table 1. The experiment was approved by the Ethics Committee of Zhejiang Normal University. Written informed consent was obtained from all participants before the test.

### 2.2. EEG Data Acquisition and Preprocessing

EEG signals were collected using the Nicolet EEG TS215605, an EEG apparatus, according to the international 10–20 system. Moreover, 16 electrodes were selected, including FP1, FP2, F3, F4, C3, C4, P3, P4, O1, O2, F7, F8, T3, T4, T5, and T6, where mastoids (A1 and A2) were used as reference electrodes. The sampling frequency of the data acquisition process was set as 250 Hz, and the electrode impedance was less than 5000 Ω. During data collection, patients were asked to rest with their eyes closed and focus on their breathing. EEG data were collected for ten consecutive minutes. The whole experiment was implemented in a professional EEG lab in Huzhou Third People’s Hospital and Shaoxing People’s Hospital.

**Figure 1:**
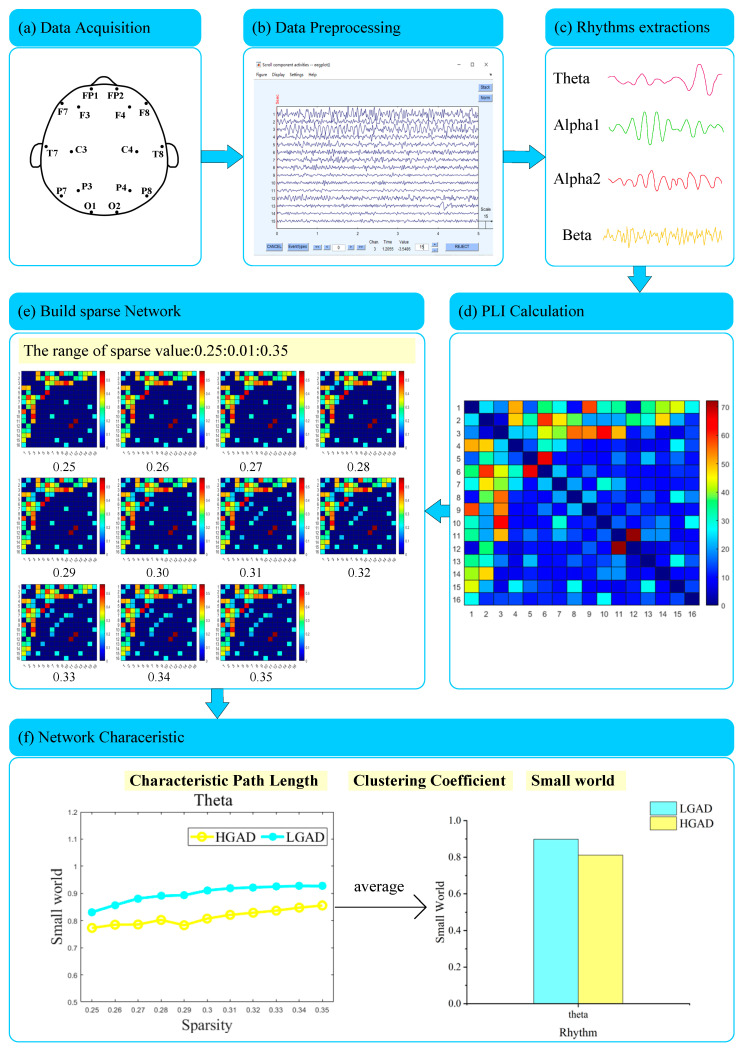
Procedures of data processing. (**a**) EEG data collection with 16 channels. (**b**) EEG data preprocessing without artifacts. (**c**) EEG rhythm extraction. (**d**) Adjacent matrix calculation with PLI values. (**e**) Functional brain network construction with the threshold value from 25% to 35% in 1% steps. (**f**) Network characteristic computations. Each adjacency matrix will obtain 11 values for each network characteristic based on 11 thresholds, which are averaged to form the final network characteristics corresponding to the adjacency matrix.

As shown in Figure 1, data preprocessing was done after the data collection was completed. Firstly, de-artifacting of EEG data was executed by fast-ICA, removing artifacts such as eyes blinking, ECG, electromyography, and so on. Secondly, the EEG data were down-sampled from 250 Hz to 125 Hz. Thirdly, high- and low-pass signals below 4 HZ and above 30 HZ were filtered out by using the fourth-order Butterworth bandpass filter. Then, 4 s of continuous EEG data with 50% overlap were applied for EEG segmentation to obtain 6587 LGAD data samples and 5012 HGAD data samples. Finally, the same bandpass filter was used to separate the EEG rhythms of theta (4–8 Hz), alpha1 (8–10 Hz), alpha2 (10–13 Hz), and beta (13–30 Hz) of each EEG sample.

### 2.3. Functional Brain Network Construction

Functional brain networks are a nonlinear dynamic analysis tool. They can be used to describe a variety of complex systems, especially suitable for describing the neurophysiological activities between different brain regions. EEG has been proven to be effective in measuring the FC between brain regions and building complex brain functional networks [38]. PLI, a widely used method of FC, was used to build functional brain networks in this study.

PLI is defined as a measure of asymmetry of the phase difference distribution between two signals [39]. It relieves the volume conduction effect on the EEG signal acquisition [40]. This is ideal for processing EEG signals. For any given two-channel EEG signals $x_{1}\left(t\right)$ and $x_{2}\left(t\right)$, PLI is calculated as follows. Firstly, we calculate the instantaneous phase $\varphi_{i}\left(t\right)$ of the $x_{i}\left(t\right)$ by the Hilbert transform [41], as in Equation (1). Here, P.V. represents the Cauchy principle value to avoid singularities where the integral falls at *τ* = *t* and *τ* = ±∞; $\left|z_{i}\left(t\right)\right|$ is the modulus of the complex number $z_{i}\left(t\right)$, and $\left|z_{i}\left(t\right)\right|e^{j\varphi_{i}\left(t\right)}$ is the exponential representation of $z_{i}\left(t\right)$ with Euler’s formula. Then, $\varphi_{1}\left(t\right)$ and $\varphi_{2}\left(t\right)$ can be obtained by Equation (1). Secondly, instantaneous phase $z_{1}\left(t\right)$ and $z_{2}\left(t\right)$ are used to calculate the phase difference between two groups of timing signals, as in Equation (2), where *arg* (denoting the argument of a complex number) denotes the principal value of the angle of the complex function, and $z_{2}^{*}\left(t\right)=\left|z_{2}\left(t\right)\right|e^{-j\varphi_{2}\left(t\right)}$ is the complex conjugate of $z_{2}\left(t\right)$.

(1) $$z_{i}\left(t\right)=x_{i}\left(t\right)+j\frac{1}{\pi }P.V.\int_{-\infty }^{\infty }\frac{x_{i}\left(t\right)}{t-\tau }d\tau =\left|z_{i}\left(t\right)\right|e^{j\varphi_{i}\left(t\right)}$$

(2) $$\Delta \varphi \left(t\right)=\varphi_{1}\left(t\right)-\varphi_{2}\left(t\right)=\arg\left(e^{j\left(\varphi_{1}\left(t\right)-\varphi_{2}\left(t\right)\right)}\right)=\arg\left(\frac{z_{1}\left(t\right)\times z_{2}^{*}\left(t\right)}{\left|z_{1}\left(t\right)\right|\left|z_{2}\left(t\right)\right|}\right)$$

Then, PLI can be defined as in Equation (3).

(3) $$PLI=\left|\langle \mathrm{sign}\Delta \varphi \left(t\right)\rangle \right|$$

where $\Delta \varphi \left(t\right)$ is the phase difference between EEG signals $x_{1}\left(t\right)$ and $x_{2}\left(t\right)$, *sign* is the signum function, $\left|•\right|$ is the symbol for calculating the absolute value, and $\langle •\rangle$ is the symbol for calculating the average value. PLI ranges between 0 and 1. The larger the PLI value, the stronger the phase synchronization of the two EEG channels.

Based on the calculation process, the PLI value was taken as the phase relation between two EEG channels. Moreover, 16 electrodes were used, with 16 × (16 − 1)/2 = 120 PLI values. At the same time, four rhythms were obtained through band-pass filtering, so there were 4 × 120 = 480 PLI values. 

The PLI values were used to construct functional brain networks. Firstly, 120 PLI values of each rhythm were selected and each edge was assigned its own PLI value according to the positions of the electrode and the edges connected to the other electrodes. The fully connected weighted network (the value of the existing connection was the corresponding PLI value) was constructed in this way. However, it was of no practical significance in the analysis to construct functional brain networks as a fully connected weighted network. Therefore, threshold ranges were set. The specific approach was to set the threshold value from 25% to 35% in 1% steps. This entailed sorting the 120 PLI values in descending order from largest to smallest, taking the top PLI values to retain based on the threshold value, deleting the rest of the PLI values, and thus building a sparse network with the retained edges, which resulted in 11 networks obtained. The principle of threshold setting was to avoid isolated nodes in sparse networks and to separate PLI values with small weights as much as possible. This was used to ensure that the differential energy of different groups of brain networks was expanded. 

### 2.4. Feature Calculation of Functional Brain Networks

The weighted characteristics of functional brain networks (including clustering coefficient, characteristic path length, and small-world attributes) were calculated from the series of 11 weighted networks. Then, the results from these 11 networks were averaged for further analysis (see Figure 1f).

#### 2.4.1. Clustering Coefficient

The weighted clustering coefficient is the probability of the connections between a node and its adjacent nodes in the network, which is generally represented by $C_{i}^{w}$ in the weighted network. The calculation of the weighted clustering coefficient was proposed by Alon et al. [42]. A higher value means more efficient information transfer. The clustering coefficient of the weighted network calculated in this study was realized by the following Formulas (1) and (2).

(4) $$C_{i}^{w}=\frac{1}{S_{i}\left(k_{i}-1\right)}\underset{i\neq j\neq h}{\sum }\frac{\left(w_{ij}+w_{ih}\right)}{2}a_{ij}a_{ih}a_{jh}$$

(5) $$C^{w}=\frac{1}{N}\overset{N}{\underset{i=1}{\sum }}C_{i}^{w}$$

where $k_{i}$ indicates the degree of node *i* in the binary network (the values of the existing connections are set to 1), $S_{i}$ represents the weighted degree of node *i* in the weighted network, $S_{i}\left(k_{i}-1\right)$ is the normalization coefficient to ensure $C_{i}^{w}$ in the range of 0 to 1, and $w_{ij}$ represents the weight value between nodes *i* and *j*. $a_{ij}$ indicates the connection between node *i* and *j*. If there is a connection, $a_{ij}=1$. If there is no connection, $a_{ij}=1$.

#### 2.4.2. Characteristic Path Length

The weighted characteristic path length is defined as the average shortest path length of all node pairs in the network, where the weighted shortest path length $l_{ij}^{w}$ describes the minimum value that node *i* to node *j* must pass through. The weighted characteristic path length was represented by $L^{w}$ in the weighted network, and it is the key parameter of information transmission and information processing in the functional brain network. The specific calculation process is shown in Equations (6) and (7).

(6) $$l_{ij}^{w}=\min\left(\frac{1}{w_{ik}}+\frac{1}{w_{kh}}+\cdots +\frac{1}{w_{mn}}+\frac{1}{w_{nj}}\right)$$

(7) $$L^{w}=\frac{1}{N\left(N-1\right)}\underset{i\neq j}{\sum }l_{ij}^{w}$$

where, in Equation (6), *k*, *h*, …, *m*, *n* refer to the nodes that the shortest path length between node *i* to node *j* may pass through, and $w_{ik}$ represents the weight value of the edge between node *i* and node *k* in the weighted network. In Equation (7), *N* represents the number of nodes in the weighted network. Here, *N* = 16.

#### 2.4.3. Small World

Small world can be used to simulate changes in the brain’s structure and function [43], and describe the process of the brain in balancing and optimizing functional separation and integration. It is positioned between general random network and regular network theory. Small world is a comprehensive index calculated using the clustering coefficient and characteristic path length. When the brain functional network satisfies Equation (8), i.e., σ>1, it indicates that the network has a small-world attribute and is an optimized network structure. Small world can be calculated by Equation (9), where $C_{\mathrm{rand}}^{w}$ and $L_{\mathrm{rand}}^{w}$ represent the weighted clustering coefficient and weighted characteristic path length in the weighted random network. The random network was generated from the experimentally obtained network by a constrained shuffle of the edges among nodes, keeping both the number of nodes and the degree distribution constant. The random networks were generated with a procedure described by Maslov and Sneppen [44].

(8) $$\left\{\begin{array}{c} C^{w}\gg C_{\mathrm{rand}}^{w}\\ L^{w}\geq L_{\mathrm{rand}}^{w} \end{array}\right.$$

(9) $$\sigma =\frac{\frac{C^{w}}{C_{\mathrm{rand}}^{w}}}{\frac{C^{w}}{L_{\mathrm{rand}}^{w}}}$$

### 2.5. Notation Interpretation in the Equations

In this section, all the symbols in Equations (1) to (9) are given for summary. $x_{i}\left(t\right)$ denotes the time series of an EEG channel. *N* represents the number of nodes, which is equal to the number of the EEG channels. $z_{i}\left(t\right)$ is the instantaneous phase of the $x_{i}\left(t\right)$. $\Delta \varphi \left(t\right)$ is the phase difference between EEG signals $x_{1}\left(t\right)$ and $x_{2}\left(t\right)$. *sign* is the signum function. $\left|•\right|$ denotes the absolute value. $\langle •\rangle$ denotes the average value. $k_{i}$ is the degree of node *i* in the binary network. $S_{i}$ is the weighted degree of node *i* in the weighted network.$\;w_{ij}$ is the weight value between nodes *i* and *j* in the weighted network. $a_{ij}$ indicates the connection between node *i* and *j* in the binary network. $C_{\mathrm{rand}}^{w}$ and $L_{\mathrm{rand}}^{w}$ represent the weighted clustering coefficient and weighted characteristic path length in the weighted random network.

### 2.6. Statistical Analysis

In order to distinguish the differences in the functional brain networks between LGAD and HGAD, one-way ANOVA was employed for the PLI values, clustering coefficient, characteristic path length, and small-world attributes of different rhythms. In addition, *p* < 0.05 was set as the obvious statistical difference.

## 3. Results

**Figure 2:**
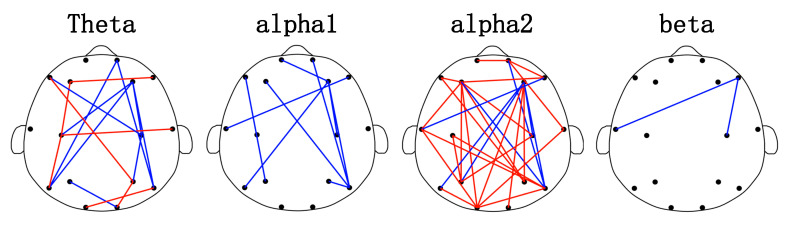
Brain function network topology of HGAD and LGAD in theta, alpha1, alpha2, beta rhythms. The red edge indicates that HGAD has a higher weight on the edge. The blue edge indicates that LGAD has a higher PLI value.

Figure 2 shows the analysis results of brain FC. The edge (the connection between two channels) represents the connection between two electrodes. Statistical differences in PLI values were verified between the HGAD and LGAD groups. In Figure 2, the blue line represents that LGAD has a higher PLI value on the edge, and the red line represents that HGAD has a higher PLI value on the edge. The topological distribution of brain networks suggests that FC is mainly associated with the frontal brain regions and are distributed between the frontal brain regions and other regions. Specifically, 73% of FC is related to the frontal area, and this ratio is 10/16, 8/9, 21/29, and 2/2 for the theta, alpha1, alpha2, and beta rhythms, respectively. PLI values of HGAD were significantly enhanced in the alpha2 rhythm (accounting for 75.86% (22/29) of the total connectivity), and mainly decreased in the theta and alpha1 rhythms. 

**Figure 3:**
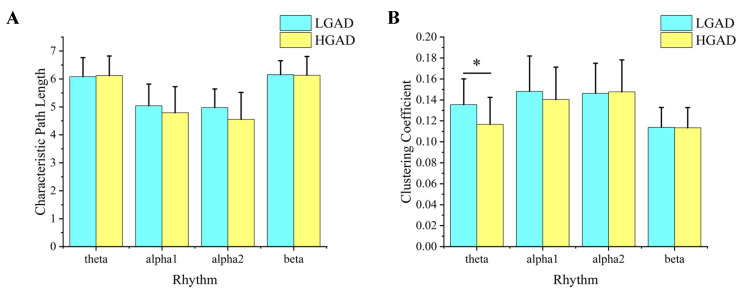
The results of network features for all EEG rhythms between LGAD and HGAD. (**A**) Characteristic path length. (**B**) Clustering coefficient. The column represents the size of the value, and * represents the statistical difference between the two groups of characteristic values at this position (*p* < 0.05).

Figure 3A,B show the average characteristic path length and average clustering coefficient of HGAD and LGAD in different rhythms (theta, alpha1, alpha2, beta). For the characteristic path length, there are no significant differences. For the average clustering coefficient, the average clustering coefficient of HGAD is larger than that of LGAD in the alpha2 rhythm. There is a significant difference between HGAD and LGAD in the theta rhythm.

**Figure 4:**
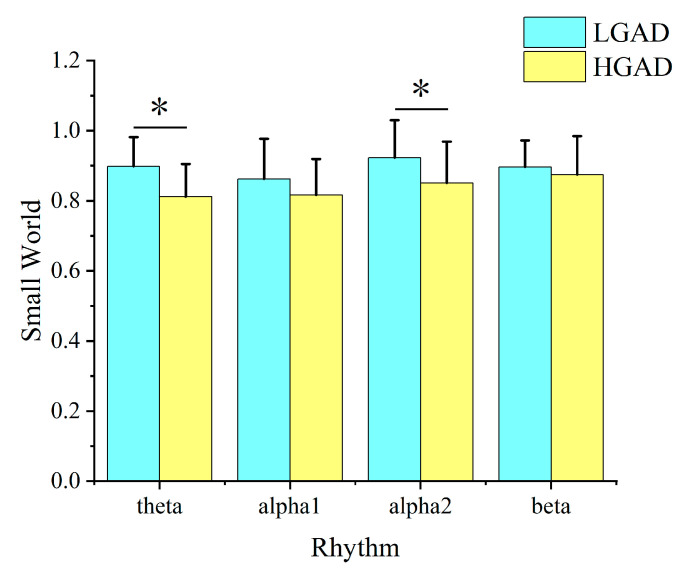
Small-world attribute analysis results of LGAD and HGAD in theta, alpha1, alpha2, and beta rhythms. Among them, the column represents the size of the value, and * represents the statistical difference between the two groups of characteristic values at this location (*p* < 0.05).

The analysis results of the small-world attributes calculated from the characteristic path length and the clustering coefficient show that the small-world value of LGAD is significantly higher than that of HGAD in the theta and alpha2 rhythms (Figure 4). Moreover, the small-world value of both groups of objects is less than 1, which does not indicate perfect small-world attributes. It should be noted that the analysis results of the characteristic path length and the clustering coefficient do not demonstrate statistical differences in the alpha2 rhythm. 

## 4. Discussion

In this resting-state EEG study, a comprehensive analysis of the brain functional networks of HGAD and LGAD was conducted. The neurodynamic mechanisms of HGAD and LGAD from the perspective of brain functional networks were analyzed. The findings are as follows. Firstly, HGAD has obviously increased values of FC in the alpha2 rhythm and completely decreased values of FC in alpha1 and decreased values of FC in the theta rhythm compared with LGAD. Moreover, the significant structural reorganization of brain functional networks is mainly related to the prefrontal brain regions. Secondly, HGAD has a lower small-world value than LGAD, which indicates that the brain functional network structure would deteriorate with the increase in GAD severity. Furthermore, both HGAD and LGAD have an unoptimized network structure. These findings will be discussed in more detail below.

### 4.1. Functional Connectivity Reorganization 

Functional connectivity refers to the ability of different brain regions to interact with each other during unconscious states [45]. By studying FC, we can understand the neural mechanisms of GAD and design better treatment plans. Our analysis showed that connections have a significant correlation with the prefrontal brain regions, distributed mostly between the frontal and other regions of the brain. A similar result has been reported by Shen et al. [4] and Song et al. [46]. Research has shown that people with generalized anxiety disorder (GAD) may have abnormal FC [47,48]. Findings from resting-state functional magnetic resonance imaging (fMRI) studies of GAD have shed light on the altered brain function and connectivity that characterize this condition [49]. Evidence of the reorganization of functional connectivity and altered functional network structure was provided in individuals with GAD. Connectivity between the amygdala and prefrontal cortex in individuals with GAD was decreased [50,51]. The amygdala is a key region involved in the processing of emotional stimuli, and the prefrontal cortex is involved in the regulation of emotional responses. Decreased connectivity between these regions may contribute to excessive anxiety and worry in individuals with GAD. Interestingly, a meta-analysis of fMRI studies in individuals with anxiety disorders, including GAD, also found decreased connectivity between the default mode network and the dorsal anterior cingulate cortex, a region involved in attentional control [52]. Individuals with GAD showed decreased connectivity within the default mode network and increased connectivity within the salience network, which is involved in the detection and integration of salient stimuli. Overall, these findings suggest that alterations in brain function and connectivity contribute to the pathophysiology of GAD [53]. The decreased connectivity between the amygdala and prefrontal cortex, as well as the alterations in functional network organization, may contribute to excessive anxiety and worry in individuals with GAD. Furthermore, the findings of decreased connectivity between the default mode network and the dorsal anterior cingulate cortex, and alterations in network structure, suggest a broader disruption of attentional control and information processing in individuals with anxiety disorders. It should be noted that the interpretation of these results is limited by the cross-sectional nature of the studies, which precludes causal inferences. Future longitudinal studies are needed to examine the development and progression of altered brain function and connectivity in individuals with GAD. Nonetheless, the current results provide a valuable insight into the neural mechanisms underlying this debilitating condition.

The reorganization of brain networks could be obtained by the changes in FC [54]. The changes in brain regional coordination and cognitive function could also be indicated by the changes in FC. Thus, in the theta rhythms and alpha1 and alpha2 rhythms, the key FC of HGAD patients showed significant reorganization throughout the brain compared to LGAD patients. There was also a similar distribution in the frontal region, which is consistent with our study. The theta rhythms have been found to be associated with increased FC between certain brain regions, such as the right frontal and central regions, and also between the right temporal and left occipital regions in individuals with GAD [55]. In patients with HGAD, reduced FC in the alpha1 rhythm may suggest disruption of inhibitory function. This disruption may impact attention and the ability to access stored information in a controlled manner. A similar result was reported in Alzheimer’s disease [56]. Compared to the theta, alpha1, and beta rhythms, the alpha2 rhythms have significantly more key FC. It accounts for 52% of all connections in all rhythms, which indicates that HGAD patients have significant whole-brain functional reorganization in the alpha2 rhythms. This may suggest that the alpha2 rhythms play a significant role in brain function and have more FC than other brain rhythms. It suggests that GAD patients show a decline in cognitive function with the severity, conforming to the general pattern of functional network changes in HGAD. However, more research is needed to fully understand the relationship between the alpha2 rhythms and FC, as well as the potential implications for brain function and various conditions. 

### 4.2. Altered Brain Functional Network Structure between HGAD and LGAD

The characteristic path length and average clustering coefficient are two indicators representing the functional integration and separation in the functional brain network, respectively [57]. In healthy individuals, the functional brain network has the best structure, with a short characteristic path length and large average clustering coefficient to maintain the balance of functional integration and separation in the brain [58]. A shorter characteristic path length indicates efficient integrity and the rapid communication of information between distant regions of the brain, which is the foundation of cognitive processing [59]. In contrast, a longer characteristic path length may reflect disruptions in the neuronal integration between regions. In the theta rhythms, the characteristic path length of HGAD is higher than that of LGAD. There is limited research on the characteristic path length in individuals with generalized anxiety disorder (GAD). A decrease in the clustering coefficient may be attributed to disruptions in functional connections between regions [60]. This suggests disarray in brain functional connections. In the theta rhythms, the significant reduction in the average clustering coefficient in the HGAD functional brain network indicates that the information processing ability in the HGAD local brain region is weakened. 

Studies have also found that individuals with various neurological and psychiatric conditions exhibit changes in their functional brain networks, including variations in small-world properties and modularity. All the values of small world in this research are less than 1. This implies that the results of GAD in reducing the small world are consistent. For all the rhythms, the small-world properties of LGAD are higher than those of HGAD. This means that the small-world features of the brain function networks of HGAD patients are weakened more. The weakness is significant in the theta and alpha2 rhythms. The decrease in small world revealed that the optimal brain functional network structure was slowly destroyed by the increasing level of GAD. The small-world attributes are decreased in GAD, indicating that the brain’s small-world network organization has been lost. Similar results have been reported in individuals with Alzheimer’s disease, schizophrenia, and depression [61]. In addition, the brain function network’s alpha2 rhythm and theta rhythm show a change to the network structure, which also reflects the reduction of the small world.

It is important to note that more research is needed to fully understand the role of changes in small world. This may aid in the development and maintenance of LGAD and HGAD to determine whether interventions that target these brain changes may be effective in treating GAD.

### 4.3. Limitations

Although the current results are meaningful, there are still some limitations in this work. Firstly, 30 HGAD patients and 21 LGAD volunteers participated in this study. This sample size is not sufficient to draw a clear conclusion. Secondly, participants were between 27 and 58 years old. A wider age range needs to be considered to obtain more comprehensive results in future studies. Thirdly, the EEG system with 16 electrodes was used in this study. Further research will focus on high-density EEG systems (e.g., 64 electrodes) and compare the results with these findings.

## 5. Conclusions

In this study, we constructed the functional brain networks of GAD and studied the characteristics of HGAD and LGAD. We found that the PLI values of HGAD were significantly increased in the alpha2 rhythm and decreased in the theta and alpha2 rhythms. The small-world value of LGAD was significantly higher than that of HGAD. In addition, the small-world values of both groups were less than 1, and the HGAD group had worse small-world attributes than LGAD. In general, this research has potential application value for the development of EEG mechanisms and diagnosis of LGAD and HGAD.

## Figures and Tables

**Table 1:** Demographic and clinical characteristics of the patients.

Characteristics	LGAD	HGAD	*p*-Value
Number	30	21	-
Age (year)	27–58 (44.90 ± 10.28)	28–58 (46.77 ± 8.99)	0.0504
HAMA	18–25 20.93 ± 2.73	29–49 35.76 ± 7.20	1.62 × 10^−13^

## Data Availability

Not applicable.

## References

[1] Oathes D.J., Ray W.J., Yamasaki A.S., Borkovec T.D., Castonguay L.G., Newman M.G., Nitschke J. (2008). Worry, generalized anxiety disorder, and emotion: Evidence from the EEG gamma band. Biol. Psychol..

[2] Wang Y., Chai F., Zhang H., Liu X., Xie P., Zheng L., Yang L., Li L., Fang D. (2016). Cortical functional activity in patients with generalized anxiety disorder. BMC Psychiatry.

[3] Saramago P., Gega L., Marshall D., Nikolaidis G.F., Jankovic D., Melton H., Dawson S., Churchill R., Bojke L. (2021). Digital Interventions for Generalized Anxiety Disorder (GAD): Systematic Review and Network Meta-Analysis. Front. Psychiatry.

[4] Shen Z., Li G., Fang J., Zhong H., Wang J., Sun Y., Shen X. (2022). Aberrated Multidimensional EEG Characteristics in Patients with Generalized Anxiety Disorder: A Machine-Learning Based Analysis Framework. Sensors.

[5] Stoychev K., Dilkov D., Naghavi E., Kamburova Z. (2021). Genetic Basis of Dual Diagnosis: A Review of Genome-Wide Association Studies (GWAS) Focusing on Patients with Mood or Anxiety Disorders and Co-Occurring Alcohol-Use Disorders. Diagnostics.

[6] Eilert N., Enrique A., Wogan R., Mooney O., Timulak L., Richards D. (2021). The effectiveness of Internet-delivered treatment for generalized anxiety disorder: An updated systematic review and meta-analysis. Depress. Anxiety.

[7] Song P.H., Tong H., Zhang L.Y., Lin H., Hu N.N., Zhao X., Hao W.S., Xu P., Wang Y.P. (2022). Repetitive Transcranial Magnetic Stimulation Modulates Frontal and Temporal Time-Varying EEG Network in Generalized Anxiety Disorder: A Pilot Study. Front. Psychiatry.

[8] Aftanas L.I., Pavlov S.V. (2005). Trait anxiety impact on posterior activation asymmetries at rest and during evoked negative emotions: EEG investigation. Int. J. Psychophysiol..

[9] Slater J., Joober R., Koborsy B.L., Mitchell S., Sahlas E., Palmer C. (2022). Can electroencephalography (EEG) identify ADHD subtypes? A systematic review. Neurosci. Biobehav. Rev..

[10] Chang Y., Stevenson C., Chen I.C., Lin D.S., Ko L.W. (2022). Neurological state changes indicative of ADHD in children learned via EEG-based LSTM networks. J. Neural. Eng..

[11] Porcaro C., Nemirovsky I.E., Riganello F., Mansour Z., Cerasa A., Tonin P., Stojanoski B., Soddu A. (2021). Diagnostic Developments in Differentiating Unresponsive Wakefulness Syndrome and the Minimally Conscious State. Front. Neurol..

[12] Ancillon L., Elgendi M., Menon C. (2022). Machine Learning for Anxiety Detection Using Biosignals: A Review. Diagnostics.

[13] Ahmad I., Wang X., Zhu M., Wang C., Pi Y., Khan J.A., Khan S., Samuel O.W., Chen S., Li G. (2022). EEG-Based Epileptic Seizure Detection via Machine/Deep Learning Approaches: A Systematic Review. Comput. Intell. Neurosci..

[14] Gadot R., Korst G., Shofty B., Gavvala J.R., Sheth S.A. (2022). Thalamic stereoelectroencephalography in epilepsy surgery: A scoping literature review. J. Neurosurg..

[15] Gao X., Lin S., Zhang M., Lyu M., Liu Y., Luo X., You W., Ke C. (2022). Review: Use of Electrophysiological Techniques to Study Visual Functions of Aquatic Organisms. Front. Physiol..

[16] Miraglia F., Vecchio F., Pappalettera C., Nucci L., Cotelli M., Judica E., Ferreri F., Rossini P.M. (2022). Brain Connectivity and Graph Theory Analysis in Alzheimer’s and Parkinson’s Disease: The Contribution of Electrophysiological Techniques. Brain. Sci..

[17] Willis P.G., Pavlova O.A., Chefer S.I., Vaupel D.B., Mukhin A.G., Horti A.G. (2005). Synthesis and structure-activity relationship of a novel series of aminoalkylindoles with potential for imaging the neuronal cannabinoid receptor by positron emission tomography. J. Med. Chem..

[18] Clegern W.C., Moore M.E., Schmidt M.A., Wisor J. (2012). Simultaneous electroencephalography, real-time measurement of lactate concentration and optogenetic manipulation of neuronal activity in the rodent cerebral cortex. J. Vis. Exp..

[19] Anders C., Arnrich B. (2022). Wearable electroencephalography and multi-modal mental state classification: A systematic literature review. Comput. Biol. Med..

[20] Sharma S., Nunes M., Alkhachroum A. (2022). Adult Critical Care Electroencephalography Monitoring for Seizures: A Narrative Review. Front. Neurol..

[21] Livint Popa L., Chira D., Dabala V., Hapca E., Popescu B.O., Dina C., Chereches R., Strilciuc S., Muresanu D.F. (2023). Quantitative EEG as a Biomarker in Evaluating Post-Stroke Depression. Diagnostics.

[22] Zhu X., Rong W., Zhao L., He Z., Yang Q., Sun J., Liu G. (2022). EEG Emotion Classification Network Based on Attention Fusion of Multi-Channel Band Features. Sensors.

[23] Arpaia P., Covino A., Cristaldi L., Frosolone M., Gargiulo L., Mancino F., Mantile F., Moccaldi N. (2022). A Systematic Review on Feature Extraction in Electroencephalography-Based Diagnostics and Therapy in Attention Deficit Hyperactivity Disorder. Sensors.

[24] Cao J., Huppert T.J., Grover P., Kainerstorfer J.M. (2021). Enhanced spatiotemporal resolution imaging of neuronal activity using joint electroencephalography and diffuse optical tomography. Neurophotonics.

[25] Stapel B., Nosel P., Heitland I., Mahmoudi N., Lanfermann H., Kahl K.G., Ding X.Q. (2021). In vivo magnetic resonance spectrometry imaging demonstrates comparable adaptation of brain energy metabolism to metabolic stress induced by 72 h of fasting in depressed patients and healthy volunteers. J. Psychiatr. Res..

[26] Li H., Zhang Q., Lin Z., Gao F. (2021). Prediction of Epilepsy Based on Tensor Decomposition and Functional Brain Network. Brain Sci..

[27] Meier J., Tewarie P., Van Mieghem P. (2015). The Union of Shortest Path Trees of Functional Brain Networks. Brain Connect..

[28] Huster R.J., Enriquez-Geppert S., Lavallee C.F., Falkenstein M., Herrmann C.S. (2013). Electroencephalography of response inhibition tasks: Functional networks and cognitive contributions. Int. J. Psychophysiol..

[29] Wang H., Chang W., Zhang C. (2016). Functional brain network and multichannel analysis for the P300-based brain computer interface system of lying detection. Expert Syst. Appl..

[30] Han C., Sun X., Yang Y., Che Y., Qin Y. (2019). Brain Complex Network Characteristic Analysis of Fatigue during Simulated Driving Based on Electroencephalogram Signals. Entropy.

[31] Yu Q., Sui J., Kiehl K.A., Pearlson G., Calhoun V.D. (2013). State-related functional integration and functional segregation brain networks in schizophrenia. Schizophr. Res..

[32] Jiao Z., Wang H., Cai M., Cao Y., Zou L., Wang S. (2020). Rich club characteristics of dynamic brain functional networks in resting state. Multimed. Tools Appl..

[33] Yuan J., Ji S., Luo L., Lv J., Liu T. (2022). Control energy assessment of spatial interactions among macro-scale brain networks. Hum. Brain Mapp..

[34] Liang Z., Chen S., Zhang J. (2022). Feature Extraction of the Brain’s Dynamic Complex Network Based on EEG and a Framework for Discrimination of Pediatric Epilepsy. Sensors.

[35] Gleiser P.M., Spoormaker V.I. (2010). Modelling hierarchical structure in functional brain networks. Philos. Trans. R. Soc. A-Math. Phys. Eng. Sci..

[36] Zhao G., Zhan Y., Zha J., Cao Y., Zhou F., He L. (2022). Abnormal intrinsic brain functional network dynamics in patients with cervical spondylotic myelopathy. Cogn. Neurodynamics.

[37] Li J., Chen J., Zhang Z., Hao Y., Li X., Hu B. (2022). A thresholding method based on society modularity and role division for functional connectivity analysis. J. Neural. Eng..

[38] Small M., Cavanagh D. (2020). Modelling Strong Control Measures for Epidemic Propagation With Networks-A COVID-19 Case Study. IEEE Access.

[39] He B., Astolfi L., Valdes-Sosa P.A., Marinazzo D., Palva S.O., Benar C.-G., Michel C.M., Koenig T. (2019). Electrophysiological Brain Connectivity: Theory and Implementation. IEEE Trans. Biomed. Eng..

[40] Stam C.J., Nolte G., Daffertshofer A. (2007). Phase lag index: Assessment of functional connectivity from multi channel EEG and MEG with diminished bias from common sources. Hum. Brain Mapp..

[41] Iakovidou N.D. (2017). Graph Theory at the Service of Electroencephalograms. Brain Connect..

[42] Moezzi B., Pratti L.M., Hordacre B., Graetz L., Berryman C., Lavrencic L., Ridding M.C., Keage H.A., McDonnell M.D., Goldsworthy M.R. (2019). Characterization of Young and Old Adult Brains: An EEG Functional Connectivity Analysis. Neuroscience.

[43] Alon N., Yuster R., Zwick U. (1997). Finding and counting given length cycles. Algorithmica.

[44] Watts D.J., Strogatz S.H. (1998). Collective dynamics of ‘small-world’ networks. Nature.

[45] Maslov S., Sneppen K. (2002). Specificity and stability in topology of protein networks. Science.

[46] Cao J., Garro E.M., Zhao Y. (2022). EEG/fNIRS Based Workload Classification Using Functional Brain Connectivity and Machine Learning. Sensors.

[47] Xiong H., Guo R.J., Shi H.W. (2020). Altered Default Mode Network and Salience Network Functional Connectivity in Patients with Generalized Anxiety Disorders: An ICA-Based Resting-State fMRI Study. Evid. Based Complement. Altern. Med..

[48] Guo X., Yang F., Fan L., Gu Y., Ma J., Zhang J., Liao M., Zhai T., Zhang Y., Li L. (2021). Disruption of functional and structural networks in first-episode, drug-naive adolescents with generalized anxiety disorder. J. Affect. Disord..

[49] De la Pena-Arteaga V., Fernandez-Rodriguez M., Silva Moreira P., Abreu T., Portugal-Nunes C., Soriano-Mas C., Pico-Perez M., Sousa N., Ferreira S., Morgado P. (2022). An fMRI study of cognitive regulation of reward processing in generalized anxiety disorder (GAD). Psychiatry Res. Neuroimaging.

[50] Dong M., Xia L., Lu M., Li C., Xu K., Zhang L. (2019). A failed top-down control from the prefrontal cortex to the amygdala in generalized anxiety disorder: Evidence from resting-state fMRI with Granger causality analysis. Neurosci. Lett..

[51] Liu W.J., Yin D.Z., Cheng W.H., Fan M.X., You M.N., Men W.W., Zang L.L., Shi D.H., Zhang F. (2015). Abnormal functional connectivity of the amygdala-based network in resting-state FMRI in adolescents with generalized anxiety disorder. Med. Sci. Monit..

[52] Mochcovitch M.D., da Rocha Freire R.C., Garcia R.F., Nardi A.E. (2014). A systematic review of fMRI studies in generalized anxiety disorder: Evaluating its neural and cognitive basis. J. Affect. Disord..

[53] Wang W., Qian S., Liu K., Li B., Sun G. (2016). Resting-state functional magnetic resonance imaging in neural mechanism of generalized anxiety disorder. Chin. J. Med. Imaging Technol..

[54] Zhong H., Wang J., Li H., Tian J., Fang J., Xu Y., Jiao W., Li G. (2022). Reorganization of Brain Functional Network during Task Switching before and after Mental Fatigue. Sensors.

[55] Dell’Acqua C., Ghiasi S., Benvenuti S.M., Greco A., Gentili C., Valenza G. (2020). Increased resting-state functional connectivity within theta and alpha frequency bands in dysphoria: Towards a novel measure of depression risk. medRxiv.

[56] Gurja J.P., Muthukrishnan S.P., Tripathi M., Sharma R. (2022). Reduced Resting-State Cortical Alpha Connectivity Reflects Distinct Functional Brain Dysconnectivity in Alzheimer’s Disease and Mild Cognitive Impairment. Brain Connect..

[57] Zhao S., Khoo S., Ng S.C., Chi A. (2022). Brain Functional Network and Amino Acid Metabolism Association in Females with Subclinical Depression. Int. J. Environ. Res. Public Health.

[58] Qiu P., Dai J., Wang T., Li H., Ma C., Xi X. (2022). Altered Functional Connectivity and Complexity in Major Depressive Disorder after Musical Stimulation. Brain Sci..

[59] Kim D.J., Bolbecker A.R., Howell J., Rass O., Sporns O., Hetrick W.P., Breier A., O’Donnell B.F. (2013). Disturbed resting state EEG synchronization in bipolar disorder: A graph-theoretic analysis. Neuroimage Clin..

[60] Zuo C., Suo X., Lan H., Pan N., Wang S., Kemp G.J., Gong Q. (2022). Global Alterations of Whole Brain Structural Connectome in Parkinson’s Disease: A Meta-analysis. Neuropsychol. Rev..

[61] Li G., Luo Y., Zhang Z., Xu Y., Jiao W., Jiang Y., Huang S., Wang C. (2019). Effects of Mental Fatigue on Small-World Brain Functional Network Organization. Neural. Plast.

